# Behavior of Leaf Meristems and Their Modification

**DOI:** 10.3389/fpls.2015.01060

**Published:** 2015-12-01

**Authors:** Yasunori Ichihashi, Hirokazu Tsukaya

**Affiliations:** ^1^RIKEN Center for Sustainable Resource ScienceYokohama, Japan; ^2^Department of Biological Sciences, Graduate School of Science, The University of TokyoTokyo, Japan; ^3^Bio-Next Project, Okazaki Institute for Integrative Bioscience, National Institutes of Natural SciencesOkazaki, Japan

**Keywords:** *Arabidopsis thaliana*, gene regulatory network, leaf development, leaf meristems, natural variation

## Abstract

A major source of diversity in flowering plant form is the extensive variability of leaf shape and size. Leaf formation is initiated by recruitment of a handful of cells flanking the shoot apical meristem (SAM) to develop into a complex three-dimensional structure. Leaf organogenesis depends on activities of several distinct meristems that are established and spatiotemporally differentiated after the initiation of leaf primordia. Here, we review recent findings in the gene regulatory networks that orchestrate leaf meristem activities in a model plant *Arabidopsis thaliana*. We then discuss recent key studies investigating the natural variation in leaf morphology to understand how the gene regulatory networks modulate leaf meristems to yield a substantial diversity of leaf forms during the course of evolution.

## Introduction

A leaf is a flat lateral organ of the stem, and grows along three-dimensional axes: proximal-distal, medial-lateral, and adaxial-abaxial axes (Steeves and Sussex, 1989). In general, the leaf consists of a leaf blade and a leaf petiole, and the leaf is composed of different cell types including epidermal cells, palisade cells, spongy mesophyll cells, and xylem/phloem cells (Esau, 1977). Because cell proliferation and cell differentiation occur concurrently during leaf development (Donnelly et al., 1999), a single leaf maintains cells in different developmental stages such as mitotic cells, differentiating cells, and endoreduplicating cells. Therefore, an elaborated spatiotemporal regulation of organ and cellular morphologies should underpin the leaf formation.

Plants acquire the bulk of their energy from light capture by leaves, and for this reason the leaf is specialized for photosynthesis, respiration, and photoperception. Leaf shape has direct consequences on the efficiency of light capture, photosynthetic carbon fixation, and gas exchange (Nicotra et al., 2011; Chitwood et al., 2012). As a result, leaf morphology must be optimized in response to variations in environmental conditions. In addition, plant leaves are equipped with an array of structural, chemical and protein-based defenses against herbivores and pathogens, which often target leaves (Agrios, 2005). These multiple functions are accomplished by the heterologous organ and cellular morphologies in a single leaf.

Due to their sessile lifestyle, plants exhibit a variety of morphological and physiological leaf traits that have allowed adaptation to different natural habitats. Indeed, leaf structural traits such as shape, size, and venation pattern, and physiological traits such as photosynthetic mechanisms are diversified in angiosperms (Flood et al., 2011; Sack et al., 2012; Tsukaya, 2014a). There are pressing research questions regarding leaf formation and variation: How do cellular activities cause leaf formation? What are the gene regulatory networks controlling leaf development? How were the gene regulatory networks altered during evolution? In this review, we highlight recent findings on the activities of leaf meristem and their gene regulatory networks in the model plant *Arabidopsis thaliana*. Knowledge gained from studies of *A. thaliana* has facilitated evolutionary developmental studies of leaf morphology, and we discuss recent investigations of the natural variation in leaf morphology.

## Leaf meristems

### What are leaf meristems?

**Figure 1:**
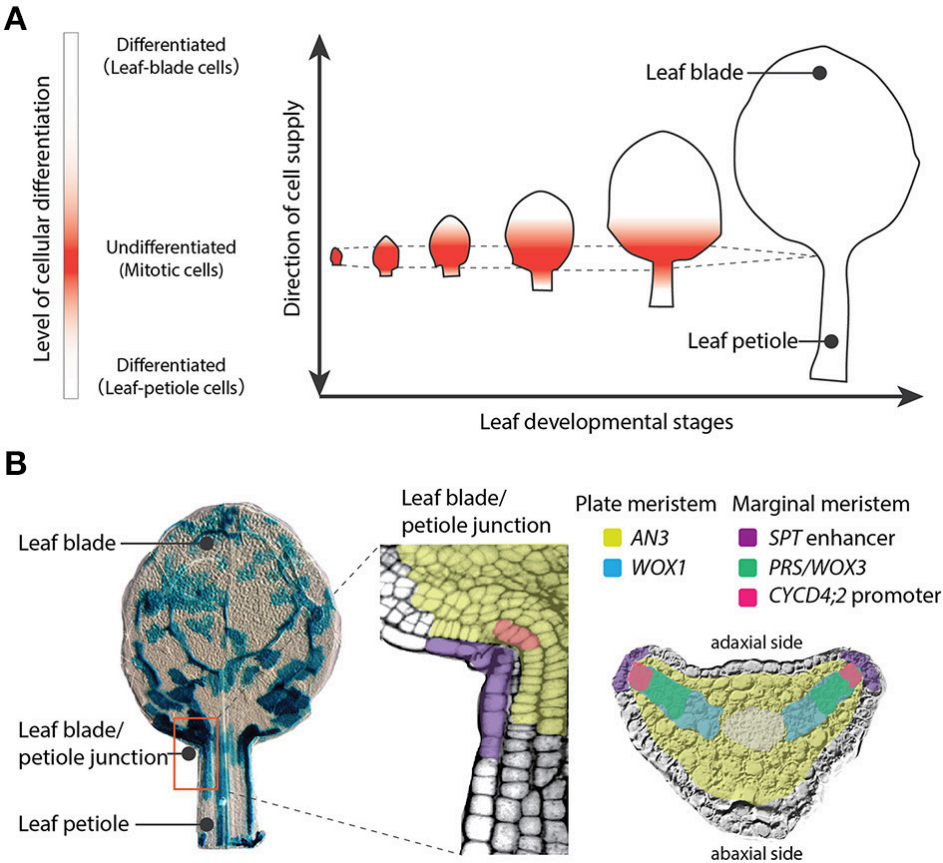
**Leaf meristems of *Arabidopsis thaliana***. **(A)** Leaf developmental stages showing the proliferative region (red). This meristematic region localizes at the leaf blade/petiole junction and produces both leaf-blade and leaf-petiole cells in a bidirectional manner. The region maintains a constant size over a limited time period. **(B)** Left image: A leaf primordium at 7 days after sowing with cell lineages indicated by blue staining (sectors were induced at 4 days after sowing). The middle and right images indicate the spatial differentiation of leaf meristems. The plate meristem is marked by *AN3* and *WOX1* gene expression domains and the marginal meristem is marked by the *SPT* enhancer, *PRS/WOX3*, and the promoter of *CYCD4;2*.

Plants have three major organs: leaves, stems, and roots. Stems and roots are directly derived from the shoot apical meristem (SAM) and the root apical meristem (RAM), respectively. The SAM and RAM maintain stem cells and exhibit indeterminate growth, which is an open-ended growth plan. On the other hand, leaves exhibit determinate growth, which is growth with a finite period of development. Meristems are defined, in a broad sense, as proliferating tissues regardless of presence of self-renewing stem cells, although the meristems have been controversial concept: Most molecular developmental biologists narrowly adopt a definition of meristems as proliferating tissues that maintain self-renewing stem cells, while meristematic tissues in leaves or stems are excluded by this definition at present because no stem cells have been observed in these tissues. (Esau, 1977; Tsukaya, 2014b). Notably WOX genes, which are key for sustaining stem cells both in SAM and RAM, are also important for the meristemactic activities in leaf primordia (Nardmann and Werr, 2013). The proliferative activity in leaf primordia is much stronger than that in the SAM, but cells that make a leaf come from restricted area of the primordium in angiosperms. Cell differentiation occurred subsequent to cell division makes the proliferative region in leaf separated spatially from SAM (Figure 1A). Therefore, leaf meristems producing leaf mesophyll cells as well as initial cells of stomata and veins are classified into intercalary meristems, that are meristematic tissues reside in a differentiating organ. Botanist Katherine Esau described in her textbook Plant Anatomy that a series of organogenesis steps in the leaf primordium depends on several distinct meristematic tissues including the plate meristem and the marginal meristem (Esau, 1977). The plate meristem consists of parallel layers of cells dividing anticlinally to play a major role in leaf growth. The marginal meristem, which is located at the edge of the leaf between the adaxial and abaxial surfaces, contributes to the establishment of tissue layers within the leaf.

These leaf meristems coordinate the orientation of the cell division plate, produce the main compartments of the leaf (the leaf blade and leaf petiole), and determine the proximal-distal gradient where the switch from cell proliferation to cell differentiation occurs in developing leaves (Donnelly et al., 1999; Ichihashi et al., 2011). This boundary is known as the cell cycle arrest front, and was assumed to progress with basipetal polarity, from the tip to the base of the leaf blade (Donnelly et al., 1999; Nath et al., 2003; White, 2006). However, recent detailed characterizations of the kinetics of cell division during leaf development have indicated that the cell-cycle arrest front does not progress gradually, but rather remains at an almost constant position, and is abolished abruptly (Kazama et al., 2010; Andriankaja et al., 2012; Figure 1A). Like the SAM or RAM, the leaf meristematic region maintains a constant size, but unlike the apical meristems, cell division ceases in the leaf after a certain time period. This leaf meristematic region is localized at the junction between the leaf blade and leaf petiole, and produces both the leaf-blade and leaf-petiole cells via cell divisions in a bidirectional manner (Ichihashi et al., 2011). In addition, leaf meristematic activity differs between tissue layers, and cell divisions directly related to the formation of veins and stomata occur throughout the period of leaf development (Donnelly et al., 1999; White, 2006; Ichihashi et al., 2011). Taken together, these studies reveal that leaves maintain their own meristems, and that the tightly controlled activity of these meristems directs the complex process of leaf tissue development.

### Regulatory mechanisms of leaf meristems

Molecular markers have been used to identify distinct regions within the leaf proliferative region of *A. thaliana*. (Figure 1B). The *ANGUSTIFOLIA3* (*AN3*) gene promoter is active in mesophyll cells just above the leaf blade/petiole junction within the leaf proliferating region (Horiguchi et al., 2005; Ichihashi et al., 2011; Kawade et al., 2013). *AN3* encodes a putative transcriptional coactivator homologous to human synovial sarcoma translocation protein, and is a positive regulator of cell proliferation in the leaf blade and leaf petiole (Kim and Kende, 2004; Horiguchi et al., 2005; Ichihashi et al., 2011). *AN3* transcripts accumulate only in mesophyll cells, but the AN3 protein moves across different leaf layers to coordinate proliferation between clonally independent leaf cells (Kawade et al., 2013). Although the exact spatiotemporal distribution of AN3 protein has to be characterized, *AN3* could mark the position of the plate meristem in leaf primordia. On the other hand, an enhancer trap line with T-DNA insertion in the 5′ region of *SPATULA* (*SPT*), along with other studies of the *SPT* promoter, show that *SPT* is expressed at the margin of the proliferative region in leaf primordia (Groszmann et al., 2010; Ichihashi et al., 2010). The promoter of a D-type cyclin gene, *CYCD4;2*, is active in a small number of cells directly adjacent to the marginal cells of the leaf primordia. Although the actual expression pattern of *CYCD4;2* is not identical to that observed in *pCYCD4;2* promoter studies (Kono et al., 2007), a specific *cis*-element seems to allow expression in this small cell population. *SPT* limits the size of the leaf proliferative region independently of *AN3* activity (Ichihashi et al., 2010), and overexpression of *CYCD4;2* promotes cell proliferation in leaves (Kono et al., 2007). Therefore, it appears that the promoter activities of *SPT* and *CYCD4;2* mark the position of the marginal meristem in leaf primordia. In addition, two WUSCHEL-RELATED HOMEOBOX (WOX) genes, *PRESSED FLOWER* (*PRS*)/*WOX3* and *WOX1*, which encode homeobox transcription factors, also redundantly promote leaf blade outgrowth (Nakata et al., 2012). *PRS/WOX3* is expressed mainly near the leaf margin (Nardmann et al., 2004) and may play a role in marginal meristem activity. *WOX1* is expressed within the two middle mesophyll layers located exactly between the adaxial and abaxial sides of the leaf blade (Nakata et al., 2012), and might be involved in plate meristem activity. Thus, leaf development depends on multiple leaf meristem activities with local controls of gene expression.

In addition to the local regulation factors, organ-level regulation of leaf meristem activity has also been identified in *A. thaliana*. *KLUH* (*KLU*) encodes the cytochrome P450 enzyme CYP78A5, which promotes organ growth, including growth of leaves, in a non-cell autonomous manner (Anastasiou et al., 2007; Adamski et al., 2009; Eriksson et al., 2010). This suggests that *KLU* is involved in generating a mobile growth factor. Computer simulation predicts that the *KLU*-dependent mobile growth factor might have less permeability or be regulated at the physical/biochemical level (Kazama et al., 2010). Computational modeling and time-lapse clonal analyses suggest that growth orientations are specified by a tissue polarity system that changes during leaf development, and that a basic pattern of growth rates across the leaf is established from an early developmental stage (Kuchen et al., 2012). Thus, organ-level regulation coordinates growth patterns at the cellular level to form leaf shape. Taken together, the studies discussed above indicate that leaf meristem activities are tightly controlled by both the local regulation systems in the plate and marginal meristem, and by mobile growth factor and tissue polarity information that functions at the organ level.

## Gene regulatory networks of leaf meristems

### Genes functioning in cell proliferation

**Figure 2:**
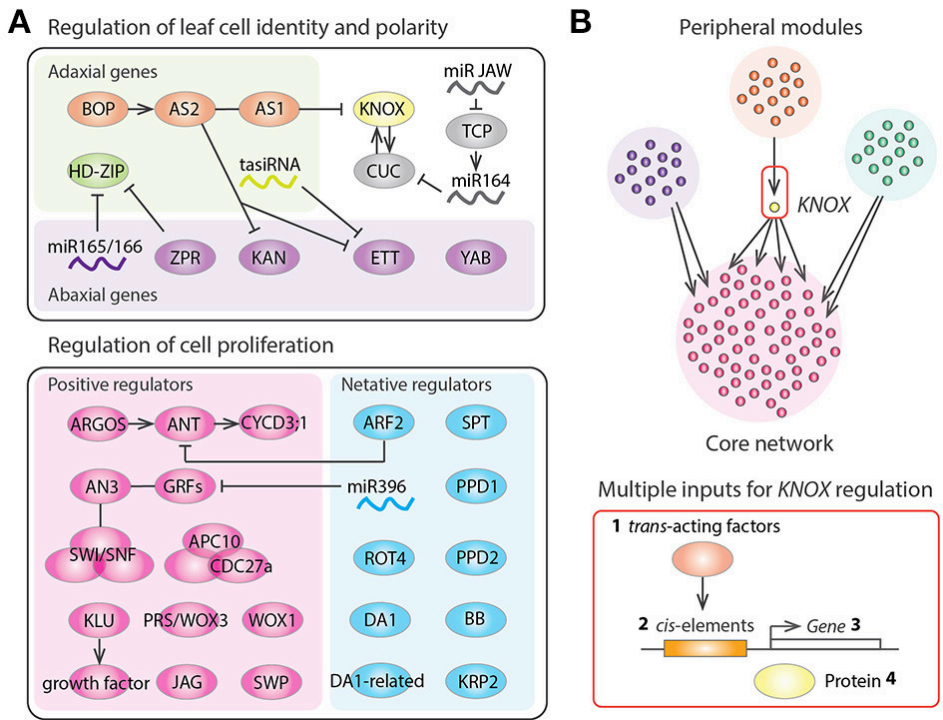
**Gene regulatory networks of leaf development**. **(A)** Regulators of leaf structural identification and leaf cell proliferation in *Arabidopsis thaliana*. Arrows, T bars, and lines indicate positive regulation, negative regulation, and protein-protein interactions, respectively. **(B)** Schematic diagram representing the gene regulatory networks controlling tomato leaf development, which consists of several peripheral gene network modules and a core network having highly interconnected genes. *KNOX* appears as a bottleneck in the network, suggesting that *KNOX* was an evolutionary hot spot that was repeatedly recruited for generating natural variation in leaf shape. *KNOX* regulation occurs at multiple levels including (1) modulation of trans-acting factors regulating *KNOX* (Ichihashi et al., 2014), (2) promoter changes at *KNOX* (Piazza et al., 2010), (3) changes in *KNOX* expression patterns (Bharathan et al., 2002), and (4) changes in effective KNOX protein concentration (Kimura et al., 2008).

A number of genes responsible for cell proliferation in leaf primordia have been identified in studies of *A. thaliana* mutants (Gonzalez et al., 2012; Kalve et al., 2014; Figure 2A). As previously mentioned, *AN3* functions at the plate meristem to produce cells of both the leaf blade and the leaf petiole (Kim and Kende, 2004; Horiguchi et al., 2005; Ichihashi et al., 2011; Kawade et al., 2013). AN3 shows protein-protein interaction with GROWTHREGULATING FACTOR5 (GRF5) to promote cell proliferation (Horiguchi et al., 2005). *AN3* is also known as *GRF-INTERACTING FACTOR1* (*GIF1*), and other members of the *GIF* family, *GIF2* and *GIF3*, also promote cell proliferation in a redundant fashion (Lee et al., 2009). AN3 binds to the SWITCH/SUCROSE NONFERMENTING (SWI/SNF) chromatin remodeling complexes to regulate transcription during leaf development (Vercruyssen et al., 2014). *AN3* is also involved in the establishment of leaf identity in cotyledons via the repression of root fate during embryogenesis (Kanei et al., 2012). On the other hands *KLU* is expressed in the basal region of leaf primordia and generates a mobile growth factor (Anastasiou et al., 2007). *PRS*/*WOX3* and *WOX1* are also classified as activators of cell proliferation (Nakata et al., 2012). The auxin inducible gene *AUXIN-REGULATED GENE INVOLVED IN ORGAN SIZE* (*ARGOS*) increases the expression level of the D-type cyclin *CYCD3;1* gene through the regulation of the *AINTEGUMENTA* genes (Krizek, 1999; Mizukami and Fischer, 2000; Hu et al., 2003; Nole-Wilson et al., 2005). APC10 and CDC27a are subunits of the anaphase-promoting complex/cyclosome (APC/C), which functions at the G2 to M transition of the cell cycle and is also reported to regulate leaf cell proliferation (Rojas et al., 2009; Eloy et al., 2011). In addition, the C2H2 zinc finger protein JAGGED (JAG) and a subunit of the Mediator complex STRUWWELPETER (SWP) are also constituent factors that positively control cell proliferation in leaves (Autran et al., 2002; Ohno et al., 2004). All of these genes function mainly in the control of lateral organ growth and not in the SAM and RAM. Therefore, a specialized set of genes is utilized to maintain leaf meristem activities.

Negative regulators of cell proliferation are important for conferring determinate growth in the leaves. As previously mentioned, *SPT* is expressed in the marginal meristem of leaf primordia to restrict the size of the leaf proliferative region (Ichihashi et al., 2010). Given that *SPT* may also help to restrict the size of the RAM (Makkena and Lamb, 2013), *SPT* might play a general role in the control of meristematic sizes in roots and leaves. MicroRNA396 represses cell proliferation through regulation of the GRF family (Jones-Rhoades and Bartel, 2004; Liu et al., 2009; Rodriguez et al., 2010). The AUXIN RESPONSE FACTOR2 (ARF2) is a repressor of auxin signaling that represses *ANT* gene expression to inhibit cell proliferation (Horiguchi et al., 2006; Schruff et al., 2006; Lim et al., 2010). The short polypeptide ROTUNDIFOLIA4 (ROT4) also functions to repress cell proliferation, especially along the proximal-distal axis (Narita et al., 2004; Ikeuchi et al., 2011; Guo et al., 2015). Therefore, a microRNA, auxin and a short peptide, all of which can potentially move across cell layers, underlie the delicate mechanisms needed to shut down leaf meristem activities. In addition, the RING-finger protein BIG BROTHER (BB); two putative ubiquitin receptors, DA and DA-RELATED (DAR); two TIFY-type transcription factors, PEAPOD1 and PEAPOD2; and the cyclin-dependent kinase inhibitor gene KIP-RELATED PROTEIN2 (KRP2) are also known to negatively regulate leaf cell proliferation (De Veylder et al., 2001; Disch et al., 2006; White, 2006; Ferjani et al., 2007; Li et al., 2008).

### Genes functioning in leaf cell identity and polarity

Several genes playing a role in the coordination of the above cell proliferation regulators have been identified in *A. thaliana* (Figure 2A). The *BLADE-ON-PETIOLE* (*BOP*) genes and their direct target *ASYMMETRIC LEAVES2* (*AS2*), which interacts with *AS1*, are involved in the recruitment of the leaf founder cells from the SAM and in the establishment of all three-dimensional axes of the leaf (Semiarti et al., 2001; Iwakawa et al., 2002, 2007; Ha et al., 2003, 2004, 2007, 2010; Xu et al., 2003; Hepworth et al., 2005; Norberg et al., 2005; Zgurski et al., 2005; Fu et al., 2007; Ikezaki et al., 2010; Jun et al., 2010; Ichihashi et al., 2011; Kojima et al., 2011; Ishibashi et al., 2012; Chen et al., 2013). *BOP* and *AS1/2* repress the expression of class I *KNOTTED-like homeobox* (*KNOX*) genes, which help maintain the indeterminate growth of the SAM (Semiarti et al., 2001; Byrne et al., 2002; Ha et al., 2003; Lin et al., 2003; Phelps-Durr et al., 2005; Guo et al., 2008). Interestingly, the chromatin-remodeling protein HIRA and the Polycomb-repressive complex2 interact with AS1/2 to bring about *KNOX* gene silencing (Phelps-Durr et al., 2005; Ueno et al., 2007; Guo et al., 2008; Lodha et al., 2013). In addition, the microRNA JAW regulates the class II TEOSINTE BRANCHED1/CYCLOIDEA/PCF (TCP) genes, which are heterochronic regulators of the leaf maturation schedule and determine the developmental window for organogenesis (Nath et al., 2003; Palatnik et al., 2003; Efroni et al., 2008). Like AN3, the TCPs also interact with SWI/SNF chromatin remodeling complex components (Efroni et al., 2013), suggesting that dynamic reorganization of chromatin architecture might play an important role throughout leaf development.

An array of genes responsible for the regulation of the adaxial-abaxial axis has been identified. These genes competitively regulate of adaxial and abaxial identity, required for flat outgrowth of the lamina. The regulators of adaxial identity are *BOP, AS1/2*, a family of class III HOMEODOMAIN LEUCINE ZIPPER (HD-ZIP) transcription factors, and a trans-acting small-interfering RNA (*tasiRNA*) (McConnell et al., 2001; Garcia et al., 2006). The regulators of abaxial identity are microRNA165/166 and *LITTLE ZIPPER* (*ZPR*), which both repress *HD-ZIP* (Mallory et al., 2004; Wenkel et al., 2007); *KANADI* (*KAN*), which is repressed by *AS2* (Wu et al., 2008); *ETTIN/ARF3*, which is repressed by *AS2* and tasiRNA (Garcia et al., 2006; Iwasaki et al., 2013; Takahashi et al., 2013). YABBY (YAB) genes also interact with the abaxial identity systems, but they are essential in switching from the SAM program to the leaf-specific program (Sawa et al., 1999; Siegfried et al., 1999; Sarojam et al., 2010). Moreover, various metabolites, ribosomal proteins, and plastid signals have been reported to affect the establishment of the leaf adaxial-abaxial axis (Pinon et al., 2008; Yao et al., 2008; Horiguchi et al., 2011; Toyokura et al., 2011, 2015; Tameshige et al., 2013). Thus, housekeeping genes working in basic metabolic and cellular functions might play specific roles in leaf development (Tsukaya et al., 2013).

In addition to three-dimensional axes of whole leaf, additional growth axes are formed to develop leaf serrations in case of *Arabidopsis thaliana*. Auxin maxima along leaf margins are required for the outgrowth of serrations, and automatically formed through the activity of auxin efflux carrier *PIN-FORMED1* (Kawamura et al., 2010; Bilsborough et al., 2011). CUP-SHAPED COTYLEDON (CUC) genes, which are generally required for boundary formation between two organs, are also key players in the serration formation (Nikovics et al., 2006). *CUC2* is essential for robustly positioning and indenting individual serrations (Bilsborough et al., 2011). *TCP* fine-tunes the expressional patterns of *CUC* through the regulation of microRNA164 to shape the serrations (Nikovics et al., 2006; Koyama et al., 2007, 2010; Kawamura et al., 2010).

## Leaf morphological evolution

Despite of the diversity in leaf morphology, the molecular mechanisms that give rise to developmental variation are incompletely understood. Evolutionary developmental biology (evo-devo) studies of plants and animals have revealed the importance of gene regulation in determining developmental variation (Blein et al., 2008; Kimura et al., 2008; Rebeiz et al., 2009; Yamaguchi et al., 2010; Loehlin and Werren, 2012). This suggests that the rewiring of developmental gene regulatory networks is a crucial causal factor driving morphological evolution (Peter and Davidson, 2011). Current evo-devo studies of plant morphology suggest that *KNOX* expression was recruited repeatedly to generate natural variation in leaf shape in several plant lineages (Bharathan et al., 2002; Kimura et al., 2008; Hay and Tsiantis, 2010; Piazza et al., 2010; Nakayama et al., 2012, 2014). Changes in the activity of other homeobox genes REDUCED COMPLEXITY also cause the repeated evolutionary modification of leaf morphology in Brassicaceae (Sicard et al., 2014; Vlad et al., 2014), suggesting that the regulation of homeobox genes was recruited repeatedly to influence leaf diversity, similar to homeobox genes contributing to animal body plan evolution (Pick and Heffer, 2012).

As we have seen, a number of leaf development genes have been identified, but the exact interactions between these genes has not been fully elucidated. The development of new genomic tools has enabled the generation of large datasets, which can be used to determine exactly how developmental gene modules are organized into a network hierarchy (Fischer and Smith, 2012). Genome-wide gene expression analyses have been performed for several plant species, to reveal the dynamic changes in gene expression during leaf development (Beemster et al., 2005; Li et al., 2010; Andriankaja et al., 2012; Ichihashi et al., 2014; Palmer et al., 2015). Ichihashi et al. (2014) used cross-species, tissue-specific, and large-scale RNA-seq data to reveal the gene regulatory networks controlling leaf development in the domesticated tomato and its wild relatives. Comparisons of the gene networks among species showed that a module regulating KNOX at the protein level had significant differences across species in a manner correlating with leaf complexity (Kimura et al., 2008; Macalister et al., 2012; Ichihashi et al., 2014). Interestingly, *KNOX* serves as a bridge connecting a peripheral gene network module to the core network that includes leaf cell proliferation regulators (Ichihashi et al., 2014). Therefore, it appears that *KNOX* is repeatedly co-opted to generate plant morphological diversity by virtue of its bottleneck location in the gene regulatory network (Figure 2B).

## Conclusion

The leaf is a complex three-dimensional photochemical reactor whose form and function are determined by gene regulatory networks. Recent technical advances are being used to unravel the mysteries of the molecular mechanisms behind leaf development and evolution. For example, gene expression studies using tissue-specific promoters will further reveal the detailed functions of leaf meristems. The key leaf development genes *KNOX, TCP*, and *AN3* are involved in epigenetic regulation (Phelps-Durr et al., 2005; Ueno et al., 2007; Guo et al., 2008; Efroni et al., 2013; Lodha et al., 2013; Vercruyssen et al., 2014). Therefore, next-generation sequencing will be useful for characterizing the genome-wide changes in chromatin structure, DNA methylation, and histone modifications during leaf development and between plant species. Novel leaf structures such as the pitcher leaves of carnivorous plants and the unifacial leaves of monocots, are generated through tissue-specific changes in cell division (Yamaguchi et al., 2010; Fukushima et al., 2015). Future studies of leaf meristem activity in determining leaf shape will undoubtedly provide greater insights into the molecular mechanisms behind the substantial diversity of leaf forms in nature.

### Conflict of interest statement

The authors declare that the research was conducted in the absence of any commercial or financial relationships that could be construed as a potential conflict of interest.
